# Burn-induced Myxedema Crisis

**DOI:** 10.5811/cpcem.2016.16.31301

**Published:** 2017-03-14

**Authors:** Ann S. Batista, Laura L. Zane, Lane M. Smith

**Affiliations:** Wake Forest School of Medicine, Department of Emergency Medicine, Winston-Salem, North Carolina

## Abstract

Myxedema crisis (MC) is a rare but life-threatening illness characterized by multi-system organ impairment from thyroid hormone deficiency that is often brought on by an eliciting event. We present the case of MC with a rapid progression of hypothermia, altered mental status, and respiratory failure that was instigated by a flash burn to the face. The patient’s condition was refractory to rewarming and supportive efforts until thyroid hormone was replaced. This case illustrates the need for a high index of suspicion for patients with a rapid onset of metabolic encephalopathy immediately after an injury or burn.

## INTRODUCTION

Myxedema crisis (MC) is a rare, life-threatening illness caused by a severe deficiency of thyroid hormone. Precipitating factors include infection, medications (withdrawal of levothyroxine, opiates, lithium, amiodarone, anesthetics, and sedatives), cerebrovascular accident, congestive heart failure, hypothermia, and trauma.^1^ The signs and symptoms of MC encompass multiple organ systems and are non-specific. They include hypothermia, generalized edema, ptosis, dry or coarse hair, fatigue, constipation, sinus bradycardia, bundle branch block, complete heart block, delayed reflex relaxation, and decreased mentation.^2^ A high index of suspicion is critical to early diagnosis since mortality approaches 60% if not quickly treated.^3^ Treatment includes supportive measures, replacing thyroid hormones, stress dose glucocorticoids, and addressing the inciting event or illness.^4^ We present a case of MC with rapid progression of hypothermia, altered mental status, and respiratory failure that was instigated by a flash burn to the face.

## CASE REPORT

A 49-year-old female poured gasoline into a running leaf blower resulting in a flash explosion that burned her face and hands. She ran from the scene into her house where she was reportedly still on fire until her family rendered first aid. She was taken to the nearest emergency department (ED) where she received a tetanus shot, wound care to her face, opiate analgesia (4 mg hydromorphone), and benzodiazepines (2 mg lorazepam) over a two-hour period. Records from the outside hospital document her as confused but normothermic at the time of arrival. She was transferred to our ED for further evaluation and management.

On arrival, she had a heart rate of 68 beats per minute, blood pressure 126/81 mm Hg, respiration rate of 7 breaths per minute, temperature 31.8 °C (89.2 °F), and an oxygen saturation of 97% on 3 liters of oxygen via nasal cannula. First- and second-degree burns were noted on her face and hands. She appeared to be in a stupor, but would awaken to verbal stimuli and follow commands with prompting. Her altered mental status and hypothermia were initially attributed to polypharmacy, and rewarming was begun with a forced-air rewarming blanket and warmed intravenous (IV) fluids. Plastic surgery evaluated her burns and recommended gentle washing with soap and water, applying bacitracin, and outpatient follow-up. However, her overall clinical picture of persistent hypothermia despite rewarming efforts and the new development of vomiting prompted additional laboratory and imaging studies. Her complete blood count, metabolic panel, lactic acid, and urinalysis were normal. An arterial blood gas showed a respiratory acidosis (7.268/55.1/136.2/24.3/-3.2). Her thyroid function reveled an elevated thyroid stimulating hormone (TSH, 25.8 μIU/ml) and depressed free thyroxine (T4) (0.8 ng/dL) consistent with hypothyroidism. A chest radiograph was obtained showing atelectasis without infiltrate, and she clinically had no evidence of pneumonia prior to being burned. Two peripheral blood cultures were sent and negative for growth at 72 hours.

Based on her elevated TSH, brief periods of bradycardia, episodes of vomiting (suggesting an ileus), respiratory failure, and history of an inciting event, she met criteria for MC. She was given 200 mcg IV levothyroxine and 100 mg IV hydrocortisone. Her mental status improved quickly with this treatment, and her temperature increased to 34.7 °C (94.5 °F) prior to being moved to her inpatient bed. She continued to improve rapidly after receiving hormone replacement with resolution of her vital sign abnormalities and respiratory failure within 24 hours of admission. She was transitioned to oral levothyroxine, and further steroids were withheld due to normal cortisol levels. She was discharged after two nights with outpatient resources for her burns and levothyroxine for hypothyroidism.

## DISCUSSION

We present the unusual case of MC brought on by severe burns to the face. MC is a rare condition with vague, non-specific clinical features that often lead to delays in diagnosis and worse outcomes.^4^ Establishing the diagnosis depends on the patient having characteristic features such as hypothermia, mental status changes, laboratory findings characteristic of hypothyroidism, and exclusion of other causes. A diagnostic scoring system exists (Table) but is rarely used due to the small number of patients from which it is derived.^5^ Scores between 20–60 are considered at risk for MC, while scores greater than 60 are considered high risk or diagnostic. This patient reached a score of 80 based on the degree of hypothermia, somnolence, presence of a precipitating event, gastrointestinal manifestations of ileus, and respiratory failure.

While trauma and burns are recognized as rare precipitating factors for MC, there are no studies defining their incidence.^6^ In addition, most patients will describe some features of hypothyroidism prior to the onset of illness, but this is not uniform.^1^ Prior to injury, the patient was healthy and without obvious symptoms of hypothyroidism, although she later recalled having abnormal thyroid function several years prior to this incident. Her presentation was rapid with severe hypothermia, brief periods of bradycardia, altered mental status, and respiratory failure developing within four hours of being burned. This suggests that she was among a small percentage of patients with compensated hypothyroidism whose symptoms manifested due to an impaired stress response.^7^

One confounding or contributing factor in this case was the amount of opiates and benzodiazepine that she received prior to arrival at our hospital. A total of 4 mg of hydromorphone was administered over a two-hour period due to severe pain prior to arrival at our facility. Further doses of opiates and other sedating substances were withheld due to her depressed mental status on arrival. While one could argue that a mixed narcotic-benzodiazepine overdose could cause hypothermia, ileus and altered mental status in the absence of hypothyroidism, we noted that her symptoms persisted for five hours after arrival and more than six hours after her last dose of sedating medications. In addition, her hypothermia was refractory to attempts at active rewarming, and only corrected when thyroid hormone was replaced. Moreover, iatrogenic overdose may explain some of her clinical features, but it would not explain the characteristic elevation in TSH and depression in her free T4. Thus, we feel that polypharmacy possibly contributed to development of MC, but does not itself account for the entire clinical picture.

Despite cases of myxedema coma being reported as early as the 1800s, very little is known about optimal treatment.^8^,^9^ Large, randomized controlled trials are hindered by the rarity of the disease; therefore, treatment recommendations are based on case series and reports as well as expert opinion. Holvey and colleagues estimated that approximately 500 mcg IV thyroxine (T4) is required to replete deficiencies seen in MC, and they demonstrated the efficacy of T4 doses ranging from 200–500 mcg in a case series of seven patients. Vital signs improved within 6–12 hours, and subjects returned to consciousness within the following 24–36 hours.^8^ Another case series of 11 patients found a trend toward lower mortality when high-dose T4 (500 mcg IV) was administered as compared to lower doses.^10^

Hydrocortisone and triiodothyroine (T3) are often given despite some controversy surrounding the use of T3 due to reports of cardiovascular complications.^9^ T4 requires conversion to the active hormone T3, and patients with severe illness have impaired conversion.^11^ Unfortunately, T3 is associated with increased mortality from cardiovascular effects in patients with advanced age, cardiac comorbidity, and high initial doses of T4 (>500 mcg) or T3 (>75 mcg).^12^ Thus, low-dose T3 (10–20 mcg) is often reserved for young patients without cardiovascular disease.^6^,^9^ In addition, adrenal insufficiency is common in patients presenting with symptoms of MC and can be difficult to distinguish from primary hypothyroidism. While there are no studies showing a clear benefit to hydrocortisone use, the potential benefit is considered to outweigh the risk.^4^

We started therapy with 200 mcg of T4 and 100 mg of hydrocortisone. A 10 mg dose of T3 was ordered but not given in the ED. Because the patient’s condition markedly improved prior to reaching her inpatient bed, the admitting team opted to withhold T3 due to the potential for cardiovascular complications associated with this drug.

## CONCLUSION

In summary, this rare case of MC brought on by burns to the face was quickly diagnosed when the clinical picture was noted to be out of proportion to the initial injury. A rapid escalation of diagnostics and therapeutics resulted in significant improvement and a favorable outcome. This highlights the need for a heightened index of suspicion in trauma and burn cases that have sudden, unexplained systemic symptoms.

## Figures and Tables

**Table:** Diagnostic scoring system for myxedema crisis

Precipitating Event	
Absent	0
Present	10

Gastrointestinal Findings	

Anorexia/abdominal pain/constipation	5
Decreased intestinal motility	10
Paralytic ileus	15

Cardiovascular Dysfunction	

Heart Rate 50–59	10
Heart Rate 40–49	20
Heart Rate <40	30
Other ECG Changes*	10
Pericardial/Pleural Effusion	10
Pulmonary edema	15
Cardiomegaly	15
Hypotension	20

Metabolic Disturbances	

Hyponatremia	10
Hypoglycemia	10
Hypoxemia	10
Present 10% Decrease in GFR	10
Hypercarbia	10

Thermoregulatory Dysfunction (°C)	

> 35	0
32–35	10
<32	20

Central Nervous System Disturbance	

Absent	0
Lethargic	10
Obtunded	15
Stupor	20
Coma/Seizures	30

*ECG*, electrocardiogram; GFR, glomerular filtration rate

* Includes: Heart blocks, Non-specific ST changes, bundle branch blocks
